# Brain Switches Utilitarian Behavior: Does Gender Make the Difference?

**DOI:** 10.1371/journal.pone.0008865

**Published:** 2010-01-25

**Authors:** Manuela Fumagalli, Maurizio Vergari, Patrizio Pasqualetti, Sara Marceglia, Francesca Mameli, Roberta Ferrucci, Simona Mrakic-Sposta, Stefano Zago, Giuseppe Sartori, Gabriella Pravettoni, Sergio Barbieri, Stefano Cappa, Alberto Priori

**Affiliations:** 1 Dipartimento di Scienze Neurologiche, Università di Milano, Milano, Italy; 2 Centro Clinico per le Neuronanotecnologie e la Neurostimolazione, Fondazione IRCCS Cà Granda - Ospedale Maggiore Policlinico, Milano, Italy; 3 Unità Operativa di Neurofisiopatologia Clinica, Fondazione IRCCS Cà Granda - Ospedale Maggiore Policlinico, Milano, Italy; 4 Unità Operativa di Neurologia, Fondazione IRCCS Cà Granda - Ospedale Maggiore Policlinico, Milano, Italy; 5 Associazione Fatebenefratelli per la Ricerca (AFaR), Ospedale “San Giovanni Calibita” Fatebenefratelli - Isola Tiberina, Roma, Italy; 6 Dipartimento di Psicologia Generale, Università di Padova, Padova, Italy; 7 Dipartimento di Scienze Sociali e Politiche, Università di Milano, Milano, Italy; 8 Centro di Neuroscienze Cognitive, Università Vita-Salute San Raffaele, Milano, Italy; 9 Dipartimento di Neuroscienze, Istituto Scientifico San Raffaele, Milano, Italy; Catholic University of Sacro Cuore, Italy

## Abstract

Decision often implies a utilitarian choice based on personal gain, even at the expense of damaging others. Despite the social implications of utilitarian behavior, its neurophysiological bases remain largely unknown. To assess how the human brain controls utilitarian behavior, we delivered transcranial direct current stimulation (tDCS) over the ventral prefrontal cortex (VPC) and over the occipital cortex (OC) in 78 healthy subjects. Utilitarian judgment was assessed with the moral judgment task before and after tDCS. At baseline, females provided fewer utilitarian answers than males for personal moral dilemmas (p = .007). In males, VPC-tDCS failed to induce changes and in both genders OC-tDCS left utilitarian judgments unchanged. In females, cathodal VPC-tDCS tended to decrease whereas anodal VPC-tDCS significantly increased utilitarian responses (p = .005). In males and females, reaction times for utilitarian responses significantly decreased after cathodal (p<.001) but not after anodal (p = .735) VPC-tDCS. We conclude that ventral prefrontal tDCS interferes with utilitarian decisions, influencing the evaluation of the advantages and disadvantages of each option in both sexes, but does so more strongly in females. Whereas cathodal tDCS alters the time for utilitarian reasoning in both sexes, anodal stimulation interferes more incisively in women, modifying utilitarian reasoning and the possible consequent actions. The gender-related tDCS-induced changes suggest that the VPC differentially controls utilitarian reasoning in females and in males. The gender-specific functional organization of the brain areas involved in utilitarian behavior could be a correlate of the moral and social behavioral differences between the two sexes.

## Introduction

“Practical issues are issues over which people are prepared to fight and kill one another; and it may be that unless some way is found of talking about them rationally and with hope of agreement, violence will finally engulf the world” [1]. Although philosophers recognized the importance of moral thinking for mankind, the biological basis of utilitarian behavior, the guiding psychological processes and underlying neurophysiological mechanisms remain unclear.

The moral judgment task is an experimental tool designed to evaluate moral reasoning by presenting various types of moral and non-moral dilemmas [2]. Subjects are required to respond to each item with a utilitarian or non utilitarian answer. In general terms, a utilitarian choice aims to obtain the maximum advantage and the minimum disadvantage, implying a personal gain, even at the expense of damaging others. Although lesional and neuroimaging studies have suggested that the ventral prefrontal cortex has a pivotal role in decisional processes [2], [3], [4], [5], [6], [7], [8], [9], [10], no data have yet shown whether interfering directly with this area, for example by applying brain stimulation, influences human utilitarian judgments. Transcranial direct current stimulation (tDCS) is a non-invasive technique for modulating brain activity [11], [12], [13], [14], [15], [16] that has already provided interesting information on decisional processes such as risk taking [17], [18], social interaction [19] and lying [20], [21].

Current evidence on how non-invasive brain stimulation influences social and non-social decision-making comes also from another brain stimulation technique widely used in neuroscience, transcranial magnetic stimulation (TMS). Repetitive TMS (rTMS) delivers short magnetic pulses that penetrate the skull and disrupt neural processing in a non-invasive way [22]. Apart from their differing underlying mechanisms, tDCS and TMS differ also in the focality of simulation: whereas TMS coils come in different sizes and configurations and can stimulate a scalp area of about 25 mm^2^, tDCS uses large electrodes measuring about 2500 mm^2^ to maintain a low current density on the scalp. Although neither tDCS nor TMS raise safety concerns, whereas rTMS is recommended for experiments investigating neurophysiological effects on specific brain circuits, tDCS is suitable for investigating modulatory effects on nonspecific neuronal populations [23]. TMS has already been used to study the role of the dorsolateral prefrontal cortex (DLPFC) in decision-making applying TMS before subjects did the Ultimatum Game [24], [25]. Right DLPFC TMS increased reaction times for rejecting unfair offers [25] and reduced subjects' rejection of their partners' intentionally unfair offers, suggesting that subjects are less able to resist the economic temptation to accept these offers [24]. Two neuroeconomic studies demonstrate also that right DLPFC TMS induces significantly riskier decisions in a gambling paradigm [26] and decreases the values assigned to food stimuli [27].

Intensive research into human decisional processes over the years shows that genders differ in moral behavior [28], [29], [30], [31], females having superior empathic ability [32], [33] and males being more prone to physical and verbal aggressiveness [34], [35]. Crime statistics report that females account for only 6–7% of inmates, irrespective of nationality, culture, religion and age [36], [37], [38].

Although previous tDCS and rTMS experiments have already linked the prefrontal cortex to fairness [19], lie [20], [21] and risk-taking behavior [17], [18], they did so using money-based tasks. Although money has a strong reward and motivational drive, human behavior is motivated also by other non-material factors, such as emotions, family affections, respect for life, non-violence, care and responsibility, fairness and equality, freedom and courage, cooperation and trust, honesty and openness. No tDCS or rTMS studies have yet investigated whether testing designed to assess non-material factors might advance our knowledge on human decision-making and behavior. Nor did previous studies on the neural basis of morality consider the important variable, gender [2], [4], [6], [7], [39]. Understanding the biological basis of utilitarian reasoning would have philosophical, sociological and neurobiological implications.

In this study we tested whether neurostimulation influencing cortical excitability in the ventral part of the anterior frontal lobe, hereafter defined as the *ventral prefrontal cortex* (VPC), elicits changes in utilitarian judgments and whether gender-related responses to tDCS underlie the gender-related differences in utilitarian thinking. To do so, in 60 healthy subjects we delivered anodal and cathodal tDCS to the VPC (VPC-tDCS). To test the specificity of our findings, in 18 subjects we then delivered tDCS also over the occipital cortex (OC-tDCS). Before and after tDCS mood was assessed using visual analog scales [40] to ensure comparable mood conditions, and participants did the moral judgment test – consisting of non moral (NM), impersonal moral (IM) and personal moral (PM) dilemmas [2] **–** to assess various material and non material factors influencing human behavior and decisions.

## Results

### Baseline Performance in the Moral Judgment Task

#### Utilitarian responses

Even though type of education (human sciences or life sciences) and religion (catholic or non catholic) had no influence on the proportion of utilitarian responses (education: p = .396, religion: p = .835, interaction: p = .301) in our sample, life sciences studies were more frequent in males than in females (58% vs. 33%; chi-square = 5.080, df = 1, p = .024) and catholic religion more frequent in females than in males (60% vs. 53%; chi-square = 1.238, df = 1, p = .266). To control for their potentially confounding effects we therefore included education and religion as covariates in the subsequent analyses.

Wald chi-square test disclosed a clear effect for type of dilemma (Wald chi-square test = 493.9; df = 2, p<.001). The post hoc sequential Sidak procedure showed that each pairwise comparison was significant (NM vs. IM, NM vs. PM and IM vs. PM: p<.001; hereafter each post hoc p-value refers to the Sidak adjustment for multiple comparisons). The Wald test also disclosed a significant interaction between “type of dilemma” and “sex” (Wald chi-square test = 6.892; df = 2, p = .032), because females and males gave similar responses for NM dilemmas (p = .965) and IM dilemmas (p = .982) but significantly dissimilar PM judgments (p = .007). At baseline males gave significantly more utilitarian responses to PM dilemmas than females (Table 1).

**Table 1:** Behavioral data pre and post tDCS.

			PRE STIMULATION						POST STIMULATION					
			% Utilitarian Responses (mean)			Mean Reaction Times (SE) in ms			% Utilitarian Responses (mean)			Mean Reaction Times (SE) in ms		
sex	stim type	stim site	NM	IM	PM	NM	IM	PM	NM	IM	PM	NM	IM	PM
F	Anodal	VPC	83%	75%	30%	4565,9 (227)	3115,9 (133)	3274,5 (136)	91%	77%	34%	4233,5 (221)	2709,3 (118)	3358,6 (224)
		OC	88%	73%	25%	4330,6 (268)	3217,9 (280)	3574,5 (267)	86%	67%	21%	3540,4 (188)	2630,4 (144)	2602,6 (157)
	Cathodal	VPC	88%	78%	24%	5069,7 (267)	3655,8 (159)	4018,9 (197)	83%	75%	25%	4238,4 (194)	3016,8 (132)	3569,1 (203)
		OC	88%	69%	31%	4402,9 (405)	3371,9 (290)	4140,7 (445)	86%	69%	40%	4103,7 (332)	3455,4 (502)	3331,3 (261)
M	Anodal	VPC	82%	71%	38%	4247,3 (223)	3513,9 (192)	4145,3 (281)	83%	77%	35%	4077,4 (230)	3130,8 (187)	3108,8 (161)
		OC	80%	67%	40%	5783,6 (594)	3655,8 (386)	4047,1 (308)	92%	67%	40%	4827,1 (468)	3074,8 (227)	4463,4 (506)
	Cathodal	VPC	87%	73%	40%	4951,9 (246)	3900,8 (206)	4594,1 (295)	85%	76%	47%	4423,6 (241)	3146,1 (182)	3650,9 (220)
		OC	92%	81%	26%	4830,4 (437)	4317,3 (327)	4753,5 (473)	87%	81%	40%	4064,7 (318)	3252,3 (454)	4156,3 (586)

F: females; M: males; SE: standard error of the mean; NM: non moral dilemmas; IM: impersonal moral dilemmas; PM: personal moral dilemmas; VPC: tDCS over the Ventral Prefrontal Cortex; OC: tDCS over the Occipital Cortex.

#### Reaction times

Similarly to utilitarian judgments, neither type of education nor religion influenced the reaction times (RTs; education: p = .438, religion: p = .972, interaction: p = .461) but given their association with sex, to control for their potentially confounding effects were entered as covariates in the subsequent analyses.

The Wald test disclosed a clear effect of type of dilemma (Wald chi-square test = 106.3; df = 2, p<.001). The post hoc sequential Sidak procedure showed that each pairwise comparison was significant (p<.001): RTs values were for NM, lowest for IM and in between for PM (mean RTs: 4726 ms, 3559 ms and 4019 ms). The Wald test also indicated that RTs could be modulated by the type of response (utilitarian or non-utilitarian). Precisely, although the p-value for “type of response” failed to reach the significance threshold of .05 (Wald chi-square = 3.458, df = 1, p = .063), the interaction “type of response x type of dilemma” was significant (Wald chi-square = 49.932, df = 2, p<.001). This interaction could be explained by computing the differences in RTs between utilitarian and non-utilitarian responses in the three types of dilemma: the difference was negative for NM (mean RTs for utilitarian responses: 4445 ms; mean RTs for non-utilitarian responses: 6380 ms; utilitarian < non-utilitarian, p<.001), non-significant for IM (mean RTs for utilitarian responses: 3451 ms; mean RTs for non-utilitarian responses: 3866 ms; utilitarian  =  non-utilitarian, p = .855) and significantly positive for PM (mean RTs for utilitarian responses: 4653 ms; mean RTs for non-utilitarian responses: 3738 ms; utilitarian > non-utilitarian, p<.001; Tables 1 and 2).

**Table 2:** Mean utilitarian and non-utilitarian reaction times pre and post tDCS.

			PRE STIMULATION						POST STIMULATION					
			Mean Utilitarian Reaction Times (SE)			Mean Non Utilitarian Reaction Times (SE)			Mean Utilitarian Reaction Times (SE)			Mean Non Utilitarian Reaction Times (SE)		
sex	stim type	stim site	NM	IM	PM	NM	IM	PM	NM	IM	PM	NM	IM	PM
F	Anodal	VPC	4122,8 (205)	3037,6 (128)	3762,1 (319)	6781,3 (765)	3348,6 (374)	3097,8 (127)	4096,7 (228)	2695,9 (136)	4642,3 (567)	5674,3 (793)	2754,1 (245)	2786,6 (152)
		OC	4205,2 (280)	3296,2 (365)	3889,5 (389)	5249,8 (872)	3002,6 (338)	3510,2 (310)	3895,58 (187)	3054 (231)	3366 (287)	4236,8 (650)	2975,9 (211)	3110,9 (195)
	Cathodal	VPC	4956,6 (285)	3578,9 (158)	5095,1 (604)	5892,1 (754)	3924,8 (457)	3706,2 (164)	3936,8 (193)	2924,5 (156)	3996,1 (509)	5688,1 (575)	3290,8 (245)	3436,3 (200)
		OC	4342,8 (452)	2927,6 (245)	5561,6 (1220)	4843,8 (716)	4355,8 (709)	3474,3 (289)	3982,9 (254)	3222,8 (353)	4557,8 (637)	6062,8 (963)	3836,2 (498)	3257,2 (172)
M	Anodal	VPC	3771,7 (176)	3213,7 (217)	4434,5 (502)	6413,9 (839)	4253 (502)	3976,2 (313)	3640,8 (179)	3125,1 (218)	3132,1 (232)	6260,2 (956)	3149,9 (377)	3140,5 (209)
		OC	5745,2 (725)	4118,1 (541)	4247,4 (525)	5937,6 (743)	2731,2 (299)	3888,6 (355)	5172,6 (422)	3614,1 (312)	5220,7 (581)	6138 (795)	2867,9 (238)	3594,1 (249)
	Cathodal	VPC	4568,1 (196)	3840,1 (229)	5462,1 (516)	7447,1 (1227)	4067,7 (456)	4027,2 (313)	4379,8 (262)	3019,4 (188)	3598,1 (303)	4677,9 (626)	3537,7 (469)	3737,1 (302)
		OC	4812,2 (463)	4061,8 (341)	3719,3 (493)	5055 (1503)	5375,6 (857)	4972 (579)	4382,4 (293)	3648,2 (312)	3311,4 (266)	5033,8 (701)	4350,5 (694)	4906,4 (502)

F: females; M: males; SE: standard error of the mean; NM: non moral dilemmas; IM: impersonal moral dilemmas; PM: personal moral dilemmas; VPC: tDCS over the Ventral Prefrontal Cortex; OC: tDCS over the Occipital Cortex.

### The Effects of tDCS on the Moral Judgment Task

#### Utilitarian responses

When pre-post tDCS changes were considered (entering “Time” as within-subjects factor), the Wald test in the 78 studied subjects disclosed a significant “Sex x Type of tDCS x Site of tDCS x Time” interaction (Wald chi-square test = 7.645; df = 2; p = .022), mainly due to the “Sex x Type of tDCS x Time” interaction for VPC (Wald chi-square = 6.255; df = 2; p = .044) and to the non-significant interaction for OC (Wald chi-square = 2.538; df = 2; p = .281).

**Figure 1:**
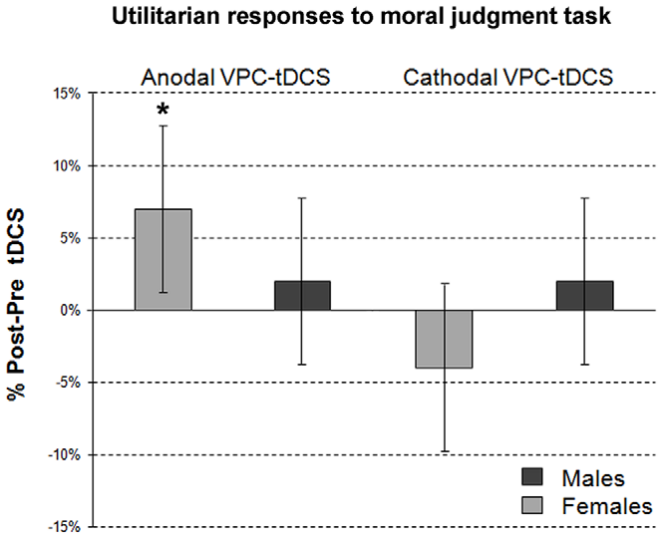
tDCS effects over the VPC on utilitarian responses to moral judgment task in males and females. Black columns: males. Gray columns: females. Y-axis: % (after-before) changes (0 = baseline) of the occurrence of utilitarian responses after tDCS. *: Wald chi-square test = 9.270; df = 2; p = .010. Error bars are the 95% confidence intervals (2 standard error of the mean). Note that whereas in males after VPC-tDCS utilitarian responses remained statistically unchanged, in females, after anodal VPC-tDCS utilitarian responses significantly increased.

The significant “Sex x Type of tDCS x Time” effect in the VPC group was not dependent on type of dilemma (“Sex x Type of tDCS x Type of dilemma x Time” interaction, Wald chi-square = 4.414, df = 4, p = .353), indicating that it can be described by collapsing the three types of dilemmas. Whereas in males neither anodal nor cathodal tDCS affected responses (Wald chi-square = 0.860; df = 2; p = .650; after anodal: p = .551, after cathodal: p = .574), in females two different patterns appeared, as indicated by the significant effect of “Type of tDCS x Time” interaction (Wald chi-square = 9.270; df = 2; p = .010): more precisely, anodal tDCS significantly increased and cathodal tDCS decreased, though not significantly, utilitarian responses (p = .005 and p = .209). In summary, VPC-tDCS changed utilitarian judgment only in females. The changes took opposite directions: anodal tDCS induced a significant increase and cathodal tDCS a non-significant decrease (Fig. 1).

The changes after OC stimulation were not significant per se (“Time” main effect, Wald chi-square = 0.236, df = 1, p = .627) nor did they differ according to type of stimulation (Wald chi-square = 2.083, df = 2, p = .353; Table 1).

#### Reaction times

Considering pre-post tDCS changes (entering “Time” as within-subjects factor), RTs were only significant per se (Wald chi-square test = 35.165; df = 1; p<.001), owing to the significant overall reduction after stimulation (from 4008 ms to 3542 ms, p<.001; Table 1). None of these changes were dependent on other considered factors; in particular, the type of response (utilitarian or non-utilitarian) had to be taken into account because it intervenes in significant double and higher–order interactions. For this reason, we ran the Generalized Estimating Equations (GEE) procedure separately for utilitarian and non-utilitarian responses.

When we investigated RTs for non-utilitarian responses, besides the overall reduction indicated by the significant “Time” effect (Wald chi-square = 14.722; df = 1; p<.001), two interactions were significant: “Sex x Type of tDCS x Time” (Wald chi-square = 7.582; df = 2; p = .023) and “Type of dilemma x Type of tDCS x Time” (Wald chi-square = 14.422; df = 4; p = .006). These effects were nevertheless non-specific insofar as the site of stimulation (VPC or OC) played no role (for each interactive term including this factor, p>.132).

Conversely, considering RTs for utilitarian responses, “Site of tDCS” was found as significant term when its interactions were evaluated. Starting with the higher order ones, the interaction “Sex x Site of tDCS x Type of tDCS x Time” was significant (Wald chi-square = 6.469; df = 2, p = .039). These data suggest that the reliable effect found in overall RTs is due to RTs for utilitarian responses. To interpret this interaction, we conducted separated analyses according to the stimulation site. Whereas RTs for utilitarian responses after OC-tDCS decreased to a similar extent after anodal or cathodal tDCS (“Type of tDCS x Time”: Wald chi-square = 0.195, df = 1, p = .659) and without further dependence on sex (“Sex x Time”: Wald chi-square = 1.261, df = 1, p = .261; “Sex x Type of tDCS x Time”: Wald chi-square = 1.392, df = 2, p = .498), RTs for utilitarian responses after VPC stimulation changed according to the time and type of tDCS (“Type of tDCS x Time”: Wald chi-square = 8.529, df = 1, p = .003). Sidak comparisons indicated that the RTs reduction was significantly larger after cathodal than after anodal tDCS (p<.001 and p = .735). Differently from the proportion of utilitarian responses, no evidence was found of sex modulation (interactive terms involving “Sex” were consistently non-significant; Table 2).

#### Subjective mood rating

Neither the mood nor happiness VAS differed between genders (Mood: F_(1,68)_ = 3.53; p = .065; Happiness: F_(1,68)_ = .10; p = .754), or differed according to the type of stimulation (Mood: F_(3,68)_ = .68; p = .579; Happiness: F_(3,68)_ = 1.42; p = .256), nor were significant differences found in the interaction “Type of tDCS x Sex” (Mood: F_(3,68)_ = .23; p = .877; Happiness: F_(3,68)_ = .56; p = .646). Hence, subjects did the moral judgment task before and after tDCS in comparable mood conditions.

## Discussion

The main finding is that neurostimulation influencing cortical excitability in the VPC elicits changes in utilitarian judgments. When we applied tDCS to the VPC but not when we applied it to the OC, we also identified distinct gender-related differences in utilitarian responses to moral as well as non-moral dilemmas. These differences might help to explain the known gender-related differences in human utilitarian reasoning. We also found a significant reduction in RTs for utilitarian responses after cathodal VPC-tDCS, regardless of type of dilemmas and of sex. These findings acquire strength because they come from a study investigating utilitarian judgments by tDCS in a large study sample, 78 subjects, balanced for sex and age and controlled for religious beliefs and type of education.

Our tDCS study using the moral judgment task to assess various material and non-material factors influencing human behavior and decisions therefore advances current knowledge on decision-making processes and utilitarian judgment.

### Baseline Performance in the Moral Judgment Task

The analyses of baseline RTs confirmed Greene et al. (2001) study, showing that utilitarian responses are slower than non-utilitarian responses specifically in PM dilemmas, but not in NM and IM dilemmas. Whereas these baseline RTs differences are independent of gender, we found significant gender-related differences in utilitarian responses studied before tDCS. These results are in line with the observation that males differ from females in cognition, decisional processes [41], [42], [43], moral judgments [28], [30] and in brain activation patterns during moral tasks [29]. These differences are independent of cultural factors, such as education levels and religious beliefs.

The gender-related differences in utilitarian responses we found before applying tDCS agree well with current knowledge. Gender-related differences in cognitive and behavioral processes are associated with functional and structural gender differences in the brain [44], [45], [46], especially in the frontal lobe [47], [48], [49], an area also involved in moral behavior [2], [3], [5], [6], [7], [9], [10]. In their study assessing altruistic cooperativeness, Yamasue et al. (2008) found that the greater cooperativeness in females correlated with larger gray matter volumes in the social brain regions such as the bilateral inferior frontal cortex. A remarkable gender-related difference has been found also in the frontal lobe neurotransmitters related to behavior [50], [51]. Finally, hormones greatly influence behavior and their receptor distribution differs between sexes in the brain structures involved with cognition [42]. The gender-related difference we found in the performance of the moral judgment task therefore fits in well with anatomical, functional, neurochemical and neuroendocrinological evidence of gender-related differences in brain areas involved in moral behavior.

### Effect of VPC-tDCS on Utilitarian Judgments

Whereas tDCS left response patterns to the moral judgment task in males unchanged, in females anodal VPC-tDCS increased the utilitarian responses for all types of dilemmas tested. Also, cathodal VPC-tDCS reduced RTs for utilitarian responses in both males and females. We therefore conclude that anodal and cathodal tDCS both interfere with rational decisions, or rational evaluation of the advantages and disadvantages of each option in both sexes, but do so more strongly in females. Our experiments indicate that tDCS-induced changes in utilitarian reasoning are site-specific and that both anodal and cathodal VPC-tDCS differentially modified subjects' performance in the moral judgment task, suggesting that the effects are specific and depend on factors other than skin perception. Equally important, the tDCS-induced changes we observed are not related to mood changes or cultural factors.

In our experiments the charge flows ventrally from the prefrontal surface to the right arm, thereby most probably stimulating the most ventral portion of prefrontal cortex. Because skull resistivity is higher than scalp resistivity, most of the current delivered by tDCS gets shunted through the scalp. The current density in the scalp also tends to decrease with the distance between electrodes. As Nathan et al. (1993) confirmed, the current density generated in the cortex by the stimulation decreases rapidly with depth – i.e. it decreases by one order of magnitude in 8 mm [52]. Also, intra-operative results show that eliciting a motor evoked potential by directly stimulating the human brainstem requires a current density of about 2–9 mA/cm^2^ [53]. For these reasons, it is unlikely that charge flows in the brainstem and structures other than the cerebral cortex below the stimulating electrode. tDCS might, however, also influence neighboring cortical areas. Even if the main effect on cortical excitability is localized beneath the stimulating electrode [54], we cannot totally exclude the possibility that tDCS also modulates other areas of the prefrontal cortex (directly, but also indirectly). Hence, notwithstanding possible tDCS-induced changes in other brain areas, we believe that tDCS induces its most important effect by modulating the VPC below the stimulating electrode.

Explaining why tDCS affects utilitarian responses in a gender-specific manner and RTs in both sexes is challenging. The female susceptibility to the effects of anodal VPC-tDCS we found in utilitarian responses could arise in several ways. For example, the gender-related effects of tDCS on utilitarian judgments might agree with the known gender specificity in the effects of cathodal and anodal tDCS on brain excitability [55], [56], [57]. Yet if they do, we find it hard to explain why none of the previous studies on the influences of brain stimulation on decisional processes reported gender-related effects [17], [18], [19].

A further more conjectural possibility is that the female tendency towards altruism is also more easily modulated by external factors. Hence whereas altruism in males is preprogrammed, in females it might be more sensitive to changes in brain plasticity. According to this hypothesis, tDCS influences utilitarian judgments in females but not in males, who responded to brain stimulation only with a reduction in RTs. This hypothesis fits in with the behavioral differences existing between genders during life: whereas in males altruistic behavior has no need to change during life, in females it has to change in relation to behavioral changes linked to reproduction and parental care [58]. This gender-related difference in altruism's sensibility to external factors is also supported by the known gender-related anatomical and functional differences in the brain structures controlling behavior and decision making. In females, a greater propensity to altruistic behavior correlates with a larger-sized inferior frontal cortex [46], so that reducing ventral prefrontal activity with tDCS could mean enhancing the cognitive and rational control of behavior.

Functional neuroimaging data with moral tasks also support a gender-related pattern of brain activation. For example, Harenski et al. (2008) found a greater activation of posterior cingulate cortex and anterior insula in females, and a greater activation of the inferior parietal cortex in males. Because the VPC is tightly linked to the cingulate cortex, tDCS over the VPC could indirectly modulate activity in the cingulate cortex. In conclusion, the differential sensitivity to tDCS in males and females could reflect gender-related anatomical, functional and neurochemical differences in the brain areas involved in utilitarian behavior. Moreover, even if gender, which also includes educational aspects, is a crucial factor for tDCS efficacy, socio-economic status and personal education might be relevant too. The relative importance of biological and social factors remains an interesting question for future researches.

A central point to clarify is how anodal and cathodal tDCS differentially modify utilitarian choices. Anodal stimulation could do so by inducing excitatory effects on the underlying cerebral cortex [11], [13], [59]. This possibility notwithstanding, even if in studies investigating tDCS-induced changes in primary cortices anodal stimulation delivered close to neurons depolarizes the neuronal membrane and cathodal stimulation hyperpolarizes it, cognitive studies leave the relation between polarity and the effects on the neuronal membrane unclear [60]. In accordance with this point of view, in our study anodal tDCS had the same effect as a lesion in the ventral portion of the frontal cortex. In their study, Koenigs et al. (2007) described lesioned patients as characterized by deficits in decision-making tasks, namely they produced an abnormally utilitarian pattern of judgments on moral dilemmas endorsing highly emotionally aversive behaviors despite an undamaged social knowledge of normative conduct [39]. Our results nevertheless showed that cathodal tDCS decreased RTs for utilitarian responses but left the proportion of utilitarian responses unchanged. Indeed, it decreased, albeit not significantly, utilitarian responses in females. These data are congruent with the study by Knoch et al. (2008) who showed that cathodal tDCS on the prefrontal cortex reduces the propensity to punish unfair behavior in the Ultimatum Game [61]. In this task, punishing unfair behavior means rejecting an unfair offer in order to obtain a fairer proposal. This is a utilitarian behavior because the subject aims to achieve a personal gain, even at the expense of damaging others. Hence cathodal and anodal tDCS both induce a functional brain system imbalance through the same mechanism. However, whereas cathodal tDCS alters the time of utilitarian reasoning in both sexes, anodal stimulation interferes more incisively, modifying utilitarian reasoning and its possible consequent actions, also in a gender-specific way. Insofar as the ventral part of the frontal lobe is phylogenetically older than the dorsolateral part [62], it might be more related to defence of the individual and to survival than to social interaction and cooperativeness.

Among other possible explanations, besides influencing cortical excitability, tDCS could induce neurochemical changes in the brain that could involve several neurotransmitters [63]. Dopamine is important for behavior, motivation and decision-making [64]. Parkinson's disease, characterized by reduced dopamine production in the brain, is associated with impaired decisional processes [65]. Anodal VPC-tDCS might therefore alter the outcomes of utilitarian decision-making processes by enhancing prefrontal dopamine. Yet, because dopamine is an anionic catecholamine that during electrophoresis migrates toward the anode, anodal VPC-tDCS could increase dopamine levels in the frontal lobe, influencing the reward circuit and ultimately altering decisional processes, increasing the rate of utilitarian responses. Interestingly, the gender-specific effects of VPC-tDCS in our experiments agree with the female-specific features of the dopaminergic system in the frontal cortex [50], [51]. Although this is a theoretical hypothesis, emerging data in humans support the interaction between tDCS and the dopaminergic system [66], [67].

The gender-related differences in utilitarian responses we found in this study using the moral judgment task in healthy subjects are a good starting-point for explaining criminal behavior. Mental illness – the inability to distinguish good from bad – reflects immoral reasoning. Our study provides evidence showing that males are by nature more utilitarian than females, and that non-invasive brain stimulation more easily and incisively alters feminine than male utilitarian thinking, thus confirming the prevalence of criminal behavior in males. Future studies designed to investigate the possibility of modulating morality and utilitarianism could better account for criminal behavior and male propensity to violate the law.

## Materials and Methods

### Subjects

Seventy-eight healthy subjects (38 men and 40 women) participated in the study (Table 3). All participants spoke native Italian, were right handed and had no history of medical, neurological, or psychiatric disorders. Also, all subjects were without acute or chronic central nervous system-affecting medication. All participants gave their written informed consent and the procedures had the approval of the ethical committee. The experimental procedure was in accordance with the declaration of Helsinki.

**Table 3:** Demographic data.

		Age	Religion		Education	
			Catholic	Non Catholic	Human Sciences	Life Sciences
**Males**	VPC-tDCS	25.7 (1.033)	53%	47%	42%	58%
	OC-tDCS	25.1 (1.407)	37.5%	62.5%	37.5%	62.5%
**Females**	VPC-tDCS	23.7 (0.573)	60%	40%	67%	33%
	OC-tDCS	22.2 (0.696)	70%	30%	70%	30%

Values are mean (standard error of the mean). VPC-tDCS: tDCS over the Ventral Prefrontal Cortex; OC-tDCS: tDCS over the Occipital Cortex.

### Transcranial Direct Current Stimulation (tDCS)

tDCS (2 mA, 15 minutes) was delivered by a constant current electrical stimulator (Eldith, Ilmenau, Germany) connected to a pair of electrodes. Two different electrode montages were used. In 60 subjects (30 males), the active electrode was placed bilaterally on forehead, just above the eyebrows, and fixed with a pair of goggles, while the reference electrode was placed over the right deltoid muscle. In this montage, because the portion of the current not shunted through the scalp enters the skull below the stimulating electrodes [68] and is directed to the lowest-impedance path towards the reference electrode, placed on the right arm [69], the charge flows ventrally from the prefrontal surface to the right arm, thereby almost certainly stimulating the most ventral portion of prefrontal cortex. In 18 volunteers (8 males), the active electrode was placed over the occipital cortex (OC). Both groups of stimulation were in turn subdivided in two groups, anodal and cathodal. Subjects were unable to distinguish anodal or cathodal tDCS. Because preliminary experiments showed that during VPC-tDCS subjects reported a mild itching sensation and a metallic taste, we did not use sham stimulation but we only compared anodal vs. cathodal tDCS.

To avoid confounding biases arising from two electrodes with opposite polarities over the scalp, we used a non-cephalic reference electrode for tDCS [20], [60], [70]. This montage allowed us to evaluate selectively the effect of scalp stimulation over a well-defined cortical area avoiding possible interference due to polarization of other cortical structures near the reference scalp electrode that can confound the source of tDCS effects. The non-cephalic reference electrode montage also yielded reproducible data during and after polarization without inducing effects related to brainstem activation [59]. The electrodes used for tDCS were thick (0.3 cm), saline-soaked synthetic sponges (scalp electrode 54 cm^2^; deltoid electrode 64 cm^2^). Because the skin on the forehead is sensitive, stimulation at 2 mA for 15 min could induce reddening and because the bone under the prefrontal electrode is thick we used a wide active electrode surface.

We ramped the current up over the first 8 s of stimulation and down over the last 8 s. To guarantee safety we applied a current at a density of 0.0037 mA/cm^2^ and delivered a total charge of 0.034 C/cm^2^. These criteria are far below the threshold for tissue damage [71]. Subjects were tested before tDCS and after it ended.

### Moral Judgment Task

We used a set of 60 dilemmas [2] translated into Italian. In accordance with Greene et al. (2001) and Fumagalli et al. (2009), these dilemmas were classified into 20 “non-moral” (NM), and two classes of “moral” scenarios subdivided into impersonal moral (IM, n = 18) and personal moral (PM, n = 22) dilemmas.

**Figure 2:**
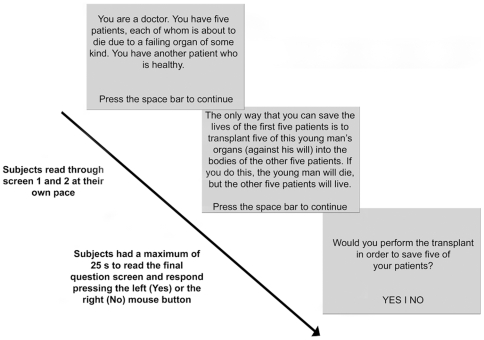
Task sequence for personal moral dilemmas. The sequence was the same for non-moral and impersonal moral dilemmas. Each dilemma was presented in a series of three screens of text. The first two screens each displayed a paragraph describing the context and details of the dilemma. The third screen posed a question about a hypothetical action related to the scenario (“Would you … in order to …?”). Participants were allowed to read through screens 1 and 2 at their own pace, pressing the space-bar to advance to the next screen. In the third screen, they were allowed no more than 25 s to read the final question screen and respond by pressing the left (yes) or the right (no) button on the mouse. In this situation, because the subject decides to sacrifice one person in order to save five persons, “Yes” is the utilitarian response that allows the maximum advantage (five alive) and the minimum disadvantage (one dead). The 60 dilemmas were randomly divided into two 30-item alternate versions of the test. These two versions were administered in a counterbalanced order, half of the subjects receiving version 1 at baseline and version 2 after tDCS, and half receiving the opposite order.

The 60 dilemmas were randomly divided into two 30-item alternate versions of the test (10 NM, 9 IM and 11 PM for each version). These two versions were administered in a counterbalanced order, half of the subjects receiving version 1 at baseline and version 2 after tDCS, and half receiving the opposite order of administration. Each dilemma was presented in a series of three screens of text. The first two screens each displayed a paragraph describing the context and details of the dilemma. The third screen posed a question about a hypothetical action related to the scenario (“Would you … in order to …?”). Participants were allowed to read through screens 1 and 2 at their own pace, pressing the space-bar to advance to the next screen. In the third screen, they were allowed no more than 25 s to read the final question screen and respond by pressing the left (yes) or the right (no) button on the mouse (Fig. 2). Subjects were instructed to respond as fast and accurately as possible. Stimuli were presented on a PC screen using E-Prime Version 1.1.

Each dilemma used could be solved by responding Yes or No, hence in each dilemma one of the two possible responses is utilitarian, whereas the other is non-utilitarian. An example of a moral dilemma is the following: *A runaway trolley is heading down the tracks toward five workmen who will be killed if the trolley proceeds on its present course. You are on a footbridge over the tracks, in between the approaching trolley and the five workmen. Next to you on this footbridge is a stranger who happens to be very large. The only way to save the lives of the five workmen is to push this stranger off the bridge and onto the tracks below where his large body will stop the trolley. The stranger will die if you do this, but the five workmen will be saved. Would you push the stranger on to the tracks in order to save the five workmen?* In this dilemma, Yes is a utilitarian response, whereas No is the non-utilitarian response. The utilitarian alternative, requiring a Yes response, consists of pushing the stranger in front of the trolley, so that even if one person is killed, five are saved. The cost-benefit analysis suggests that the most overall utility is to sacrifice one person rather than five. This response is classified as utilitarian because, according to Greene et al. (2001, 2002, 2004) and Koenigs et al. (2007), it allows subjects to maximize aggregate welfare, often overcoming the emotional response against inflicting direct harm to another person.

**Figure 3:**
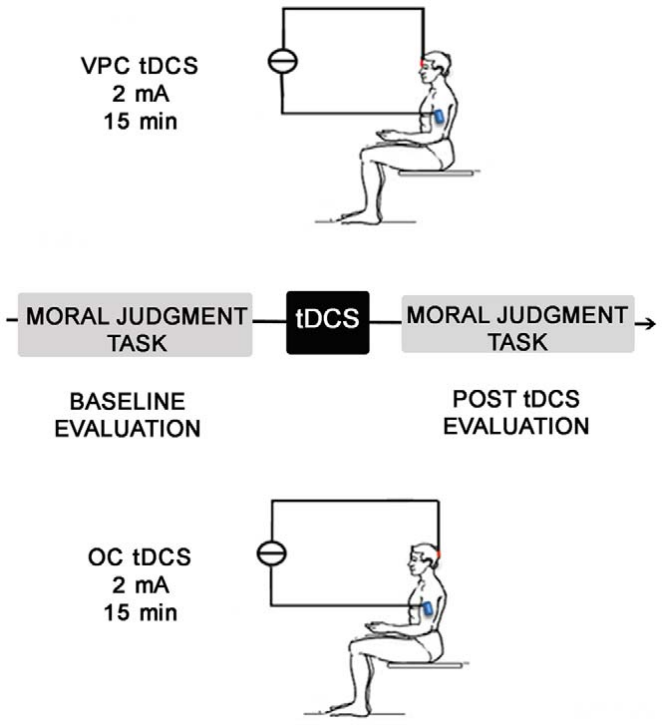
Experimental protocol. Subjects performed the moral judgment task before and after transcranial direct current stimulation (tDCS, 15 minutes, 2 mA) over the ventral prefrontal cortex (VPC-tDCS) or over the occipital cortex (OC-tDCS).

After an initial practice run, consisting in four examples of non-moral scenarios to familiarize subjects with the task, subjects were tested before tDCS and thereafter. Subjects were randomly subdivided into four groups: anodal and cathodal VPC-tDCS and anodal and cathodal OC-tDCS (Fig. 3).

### Subjective Mood Rating

To control the influence of tDCS on mood, before and after tDCS we administered two 100-mm visual analog scales [40]: a mood VAS (0 corresponding to good mood and 100 corresponding to bad mood) and a happiness VAS (0 corresponding to sadness and 100 corresponding to happiness). Subjects were asked to describe their current affective condition by marking the lines [72].

### Data Analysis

The database was constructed with information on subjects' responses for each dilemma. For each item, the response of a given subject, a given stimulation (anodal, cathodal VPC-tDCS and anodal, cathodal OC-tDCS) and a given time (pre, post tDCS) was coded as 0 (non-utilitarian) or 1 (utilitarian) and the corresponding RT was entered.

We used the GEE model in line with Koenigs et al. [39] study because this model allowed us to analyze behavioral data fully. It also avoided wasting information given by the single response and the single RT to a given item and, instead of aggregate data computing the proportion of utilitarian responses or the mean of RTs, we analyzed the whole dataset with a unified statistical approach. In particular, GEE models allowed us to assess the significance of main and interactive effects of ‘Type of tDCS’, ‘Site of tDCS’, ‘Gender,’ ‘Type of dilemma’ and ‘Time’ as predictors (factors) on utilitarian responses and RTs measures, taking into account within-subject dependence. Specifically, a binary logistic function was used to analyze the utilitarian responses as dependent variables. Conversely, when RTs were the dependent variable, a Gaussian distribution was assumed, with a log link function to obtain a better fit to Gaussianity (typically and also in our study, RTs follows an almost log-normal distribution).

First, we analyzed data obtained before stimulation. When RTs were the dependent variable, the binary variable “utilitarian responses” was entered as an additional factor because RTs could change according to utilitarian/non-utilitarian responses as well as to the interaction between “utilitarian responses” and the other potential predictors.

To assess the effect of tDCS, the “Time” factor was added to the model and its main effect as well its interaction with the other factors and covariates were evaluated. Because the subjects were also classified by their type of education (all being graduated, the major distinction was between life sciences and human sciences) and by their religion (catholic vs. non catholic), the model took into account potential confounding from these two factors.

To assess the effect of tDCS on mood, we computed the differences (after-before tDCS) and tested them in a three-way within subjects analysis of covariance (ANCOVA) with tDCS (anodal, cathodal VPC-tDCS and anodal, cathodal OC-tDCS) and gender (males, females) as factors and education and religion as covariates for mood VAS and happiness VAS.
